# Model-free predictive control for PMSM based on a nonlinear autoregressive exogenous model and an adaptive recursive least squares algorithm

**DOI:** 10.1371/journal.pone.0354803

**Published:** 2026-07-27

**Authors:** Xiaolei Shi

**Affiliations:** School of Artificial Intelligence, Anshan Normal University, Anshan, Liaoning, China; National Institute of Technology, India (Institute of National Importance), INDIA

## Abstract

This paper proposes a novel nonlinear autoregressive with exogenous input-based adaptive recursive least squares model-free predictive control (NARX-ARLS-MFPC) strategy for permanent magnet synchronous motor (PMSM) drives. The core challenge addressed is the performance degradation of conventional model predictive control (MPC) under inevitable motor parameter mismatches. The proposed method integrates the NARX model with an ARLS algorithm featuring a variable forgetting factor to dynamically track system changes. Comprehensive simulation studies validate the superior robustness of the strategy. Under significant inductance and flux linkage mismatches, the proposed method reduces current total harmonic distortion (THD) by 28.3% and 12.3% compared to conventional finite-control-set model predictive control (FCS-MPC) and a baseline control method, respectively. It maintains stable performance under moderate sensor noise with appropriate tuning. During load transients combined with resistance and inductance mismatches, it achieves THD reductions of 24.1% and 15.7% versus the two benchmark methods, respectively. Statistical analysis under parameter perturbations confirms its overall superior performance across key dynamic and steady-state metrics. The results demonstrate that the synergistic integration of the nonlinear model and adaptive identification effectively suppresses current harmonics caused by model inaccuracies while enhancing dynamic performance.

## 1. Introduction

PMSMs are widely used in modern industrial systems due to their high power density, efficiency, and reliability. Consequently, optimizing PMSM control strategies has attracted significant research interest [1–4]. To achieve high-performance control of PMSMs, various advanced control paradigms have been developed, such as sliding mode control, fuzzy control, robust control, finite-time control, and MPC [5]. Among various strategies, MPC has been widely adopted for power inverters due to its fast dynamic response, precise transient control, and superior handling of nonlinear constraints [6–10]. However, the performance of MPC is highly dependent on model accuracy. In practice, parameter mismatches and model uncertainties—caused by manufacturing tolerances, thermal variations, or unmodeled dynamics—can substantially degrade control performance and robustness [11]. To address this limitation, model-free predictive control (MFPC) techniques, which build predictive models directly from operational data rather than physical parameters, have gained attention. Prominent data-driven approaches include: lookup-table-based schemes, which map voltage vectors to current deviations [12]; methods employing an ultra-local model for system dynamics [13–15]; frameworks integrating autoregressive with exogenous input (ARX) models [16–18]; and neural-network-based schemes, which utilize reinforcement learning to achieve high-accuracy predictions [19,20]. A comparative analysis of these state-of-the-art model structures is detailed in Table 1.

**Table 1:** Comparison of prevalent data-driven MFPC methods.

Model structure	Identification/ Update method	Robustness Scenarios Evaluated	Performance Metrics Reported	Stability Analysis	Computational Burden
Lookup table model [12]	Direct update of a single lookup table entry per control cycle	Load and inductance variations, time-varying DC-link voltage	Current THD, alternating current ripple, average switching frequency	No explicit theoretical stability proof. The stagnation effect may compromise stability.	Very low: mainly memory access and simple updates.
Ultra-local Model [13–15]	Observer-based method and algebraic algorithm	Primarily motor parameter mismatches (e.g., resistance and inductance)	Current THD, settling time, speed and current tracking error, average switching frequency	Observer error convergence/ No explicit theoretical stability proof	Low to moderate: cost depends on observer and estimator complexity.
ARX model [16–18]	RLS, normalized least-mean-square, and recursive gradient correction	Uncertainties in system model structure and parameters (e.g., flux linkage, resistance, inductance mismatches, and load disturbance)	Current THD, current tracking error, average switching frequency	Relies on the convergence of the identification algorithms	Moderate: dominated by RLS-based update of an n × n covariance matrix.
Neural network model (RNN [19], ZNN [20])	Reinforcement learning (offline/online training with critic-actor networks); Extended Kalman filter or differential equation-based online update	Tested under speed step, load torque step, and noise injection	Current tracking error, convergence time	Lacks rigorous mathematical theory to guarantee stability of the network structure.	High: involves numerous multiply-accumulate operations per layer.

As shown in Table 1, recent MFPC methods have made significant progress in improving parametric robustness. A recent review [21] systematically summarizes the research progress of distributed model predictive control in unmanned aerial vehicles and vehicle platoon systems and points out that robustness under model uncertainty is still a key challenge for practical applications. Indeed, the analysis of these categories reveals that existing methods still face several common challenges: (i) a trade-off between nonlinear dynamic characterization and computational burden: for instance, ultra-local models aggregate nonlinearities into a single term, potentially losing dynamic details; neural network models impose a heavy computational burden; and (ii) limitations in adaptability and theoretical foundation under complex conditions, as some methods lack rigorous analysis frameworks for time-varying dynamics.

Among data-driven MFPC approaches, the ARX model has been successfully integrated into MFPC, as evidenced by studies such as [16–18], enhancing robustness through online parameter identification via RLS. Although effective, conventional ARX-based methods rely on a linear model structure, which inherently limits their capacity to accurately capture the nonlinear, coupled, and time-varying dynamics of PMSMs.

Unlike recent ARX-based MFPC approaches [16–18] which rely on linear ARX models, the proposed NARX-ARLS-MFPC method combines nonlinear regression with adaptive forgetting and Lyapunov-guaranteed stability, as demonstrated in Sections 3 and 4. This integration enables precise modeling of PMSM nonlinearities while ensuring robust convergence, directly addressing the limitations of prior schemes.

The proposed strategy addresses this by integrating a NARX model for nonlinear dynamic representation with an ARLS algorithm that utilizes an adaptive forgetting factor for online identification. This co-design ensures an effective trade-off between modeling accuracy and computational efficiency. Simulations under various disturbances—including parameter mismatches, variable noise levels, and sudden load changes—confirm that the NARX-ARLS-MFPC method outperforms comparative approaches, demonstrating enhanced robustness, reduced current THD, and lower steady-state error.

The main contributions of this study are summarized as follows:

- A nonlinear dynamic modeling framework: a NARX model incorporating the nonlinear coupling terms of electrical angular velocity and rotor angular position is constructed. This model provides a more accurate representation of PMSM dynamics compared to linear ARX structures.
- An ARLS algorithm with an adaptively tuned forgetting factor is employed for online identification. Furthermore, the closed-loop stability of the entire system under this scheme is analyzed and proven using Lyapunov stability theory.
- Comprehensive validation of robustness is conducted. The proposed data-driven solution is validated to maintain robust performance under a wide range of complex conditions, including severe parameter mismatches (resistance, inductance, and flux linkage), variable noise levels, and simultaneous load transients with parameter perturbations, demonstrating superior performance.

## 2. Mathematical model and system identification

### 2.1. Mathematical model of PMSM

For a surface-mounted permanent magnet synchronous motor, the mathematical model in the two-phase α-β stationary coordinate frame is described by the following complex-vector equation [20]:


$$\begin{array}{c} \frac{di_{\text{s}}}{dt}=\frac{\text{1}}{L_{\text{s}}}\left(u_{\text{s}}-R_{\text{s}}i_{\text{s}}-j\omega_{\text{e}}\psi_{\text{r}}\right) \end{array}$$ (1)

where the subscript ‘s’ denotes quantities in the stator reference frame (i.e., the α or β axis). Here, *L*_s_ is the stator inductance, ***u***_s_ is the stator voltage vector, *R*_s_ is the stator resistance, and ***i***_s_ is the stator current vector. The rotor flux linkage vector is given by ***Ψ***_r_ = *Ψ*_f_*e*^*jθ*e^, where *Ψ*_f_ is the permanent magnet flux linkage, and *θ*_e_ is the electrical angular position. The electrical angular velocity *ω*_e_ is defined as *ω*_e_ = *dθ*_e_/*dt*.

Applying a first-order discretization of Eq (1) yields the predicted current at (*k* + 1)th time instant:


$$\begin{array}{c} i_{\text{s}}^{p}\left(k+1\right)=i_{\text{s}}\left(k\right)+\frac{T_{\text{sc}}}{L_{\text{s}}}\left[u_{\text{s}}\left(k\right)-R_{\text{s}}i_{\text{s}}\left(k\right)-j\omega_{\text{e}}\psi_{\text{r}}\left(k\right)\right] \end{array}$$ (2)

where *T*_sc_ is the sampling period. Although this model enables straightforward prediction, its accuracy is highly sensitive to the precise knowledge of parameters *L*_s_, *R*_s_, and *Ψ*_f_, which are subject to variation and uncertainty in practice. This sensitivity to parametric uncertainty motivates the strategy developed in the following section.

### 2.2. ARX model

In the α-β stationary coordinate system, the input-output relationship of an unknown system can be represented using the ARX structure as follows [6]:


$$\begin{array}{c} \begin{array}{c} {\hat{i}}_{\alpha }\left(k\right)=\frac{B^{\alpha \alpha }\left(z^{-1}\right)}{A^{\alpha }\left(z^{-1}\right)}u_{\alpha }\left(k\right)+\frac{B^{\alpha \beta }\left(z^{-1}\right)}{A^{\alpha }\left(z^{-1}\right)}u_{\beta }\left(k\right)\\ {\hat{i}}_{\beta }\left(k\right)=\frac{B^{\beta \alpha }\left(z^{-1}\right)}{A^{\beta }\left(z^{-1}\right)}u_{\alpha }\left(k\right)+\frac{B^{\beta \beta }\left(z^{-1}\right)}{A^{\beta }\left(z^{-1}\right)}u_{\beta }\left(k\right) \end{array} \end{array}$$ (3)

where ${\hat{i}}_{\alpha }\left(k\right)$ and ${\hat{i}}_{\beta }\left(k\right)$ are the estimated stator currents at the k-th sampling instant; *u*_α_(*k*) and *u*_β_(*k*) are the inverter output voltages. The expressions for *A*^α^, *A*^β^, *B*^αα^, *B*^αβ^, *B*^βα^, and *B*^ββ^ are as follows:


$$\begin{array}{c} \begin{array}{c} A^{\text{i}}\left(z^{-1}\right)=1+a_{\text{1}}^{\text{i}}z^{-1}+a_{\text{2}}^{\text{i}}z^{-2}+\cdots +a_{n_{A}}^{\text{i}}z^{-n_{A}}\\ B^{\text{ij}}\left(z^{-1}\right)=b_{\text{1}}^{\text{ij}}z^{-1}+b_{\text{2}}^{\text{ij}}z^{-2}+\cdots +b_{n_{B}}^{\text{ij}}z^{-n_{B}} \end{array} \end{array}$$ (4)

where the superscript i of the polynomials *A*^i^(*z*^-1^) and *B*^ij^(*z*^-1^) denotes the axis (α or β) of the modeled current, and j denotes the axis (α or β) of the external input voltage. For simplicity, the orders of all *A*^i^(*z*^-1^) polynomials are set to *n*_*A*_ and the orders of *B*^ij^(*z*^-1^) polynomials are set to *n*_*B*_. For instance, when *n*_*A*_ = 3, *n*_*B*_ = 2, the unknown parameter vectors to be identified in Eq (3) and Eq (4) form the vectors ***η***_α_(*k*)∈ℝ^7×1^ and ***η***_β_(*k*)∈ℝ^7×1^:


$$\begin{array}{c} \begin{array}{c} \eta_{\alpha }\left(k\right)={\left[a_{\text{1}}^{\alpha }\left(k\right),\text{\textbackslash{}hspace\{0.33em}\}a_{\text{2}}^{\alpha }\left(k\right),\text{\textbackslash{}hspace\{0.33em}\}a_{\text{3}}^{\alpha }\left(k\right),b_{\text{1}}^{\alpha \alpha }\left(k\right),\text{\textbackslash{}hspace\{0.33em}\}b_{\text{2}}^{\alpha \alpha }\left(k\right),\text{\textbackslash{}hspace\{0.33em}\}b_{\text{1}}^{\alpha \beta }\left(k\right),\text{\textbackslash{}hspace\{0.33em}\}b_{\text{2}}^{\alpha \beta }\left(k\right)\right]}^{T}\\ \eta_{\beta }\left(k\right)={\left[a_{\text{1}}^{\beta }\left(k\right),\text{\textbackslash{}hspace\{0.33em}\}a_{\text{2}}^{\beta }\left(k\right),\text{\textbackslash{}hspace\{0.33em}\}a_{\text{3}}^{\beta }\left(k\right),b_{\text{1}}^{\beta \alpha }\left(k\right),\text{\textbackslash{}hspace\{0.33em}\}b_{\text{2}}^{\beta \alpha }\left(k\right),\text{\textbackslash{}hspace\{0.33em}\}b_{\text{1}}^{\beta \beta }\left(k\right),\text{\textbackslash{}hspace\{0.33em}\}b_{\text{2}}^{\beta \beta }\left(k\right)\right]}^{T} \end{array} \end{array}$$ (5)

Their corresponding regression vectors ***φ***_α_(*k*)∈ℝ^7×1^ and ***φ***_β_(*k*)∈ℝ^7×1^, composed of the historical input and output data, are given by:


$$\begin{array}{c} \begin{array}{c} \varphi_{\alpha }\left(k\right)={\left[\begin{array}{ccc} @ccc@i_{\alpha }\left(k-1\right), & i_{\alpha }\left(k-2\right), & i_{\alpha }\left(k-3\right),\text{\textbackslash{}hspace\{0.33em}\}u_{\alpha }\left(k-1\right), \end{array}\text{\textbackslash{}hspace\{0.33em}\}u_{\alpha }\left(k-2\right),\text{\textbackslash{}hspace\{0.33em}\}u_{\beta }\left(k-1\right),\text{\textbackslash{}hspace\{0.33em}\}u_{\beta }\left(k-2\right)\right]}^{T}\\ \varphi_{\beta }\left(k\right)={\left[\begin{array}{ccc} @ccc@i_{\beta }\left(k-1\right), & i_{\beta }\left(k-2\right), & i_{\beta }\left(k-3\right),\text{\textbackslash{}hspace\{0.33em}\}u_{\alpha }\left(k-1\right), \end{array}\text{\textbackslash{}hspace\{0.33em}\}u_{\alpha }\left(k-2\right),\text{\textbackslash{}hspace\{0.33em}\}u_{\beta }\left(k-1\right),\text{\textbackslash{}hspace\{0.33em}\}u_{\beta }\left(k-2\right)\right]}^{T} \end{array} \end{array}$$ (6)

The RLS algorithm is then employed to estimate the parameter vectors ***η***_α_(*k*) and ***η***_β_(*k*).

### 2.3. RLS parameter estimation

The parameter estimation process based on the RLS algorithm is described below, taking the α-axis as an example. The cost function *J*(*k*) based on the least-squares criterion is given as follows:


$$\begin{array}{c} \underset{\eta_{\alpha }\left(k-1\right)}{min}J\left(k\right)=\frac{\text{1}}{\text{2}}\sum_{l=1}^{k}\lambda^{k-l}{\|i_{\alpha }\left(l\right)-\varphi_{\alpha }^{T}\left(l\right)\eta_{\alpha }\left(k-1\right)\|}^{\text{2}} \end{array}$$ (7)

where *λ* (0 < *λ* ≤ 1) is the forgetting factor. The summation collects the squared errors between the measured current *i*_α_(*l*) and the mode*l* prediction ***φ***_α_^*T*^(*l*)***η***_α_(*k*-1) over a*l*l past sampling instants *l* = 1, 2, …, *k*. The RLS a*l*gorithm that minimizes *J*(*k*) is given by the following recursive equations:


$$\begin{array}{c} \begin{array}{c} {𝒢}_{\alpha }\left(k\right)={𝒫}_{\alpha }\left(k-1\right)\varphi_{\alpha }\left(k\right){\left[\lambda +\varphi_{\alpha }^{\text{T}}\left(k\right){𝒫}_{\alpha }\left(k-1\right)\varphi_{\alpha }\left(k\right)\right]}^{-1}\\ {\hat{\eta }}_{\alpha }\left(k\right)={\hat{\eta }}_{\alpha }\left(k-1\right)+{𝒢}_{\alpha }\left(k\right)e_{\alpha }\left(k\right)\\ {𝒫}_{\alpha }\left(k\right)=\lambda^{-1}\left[ℐ-{𝒢}_{\alpha }\left(k\right)\varphi_{\alpha }^{\text{T}}\left(k\right)\right]{𝒫}_{\alpha }\left(k-1\right) \end{array} \end{array}$$ (8)

Here, ***P***_α_(*k*) is the covariance matrix, ***G***_α_(*k*) is the gain matrix, and the current estimation error *e*_α_(*k*) is expressed as:


$$\begin{array}{c} e_{\alpha }\left(k\right)=i_{\alpha }\left(k\right)-\varphi_{\alpha }^{\text{T}}\left(k\right){\hat{\eta }}_{\alpha }\left(k-1\right) \end{array}$$ (9)

## 3. MFPC method based on NARX and ARLS

### 3.1. NARX model

To better capture the nonlinear characteristics of PMSM, particularly the back-EMF effects, specific nonlinear terms involving the electrical angular speed and the rotor angular position are incorporated into the regression vector. This enhancement, along with a simplification to reduce model order, leads to the following compact NARX regression vectors:


$$\begin{array}{c} \begin{array}{c} \varphi_{\alpha }\left(k\right)={\left[\begin{array}{c} @ccc@i_{\alpha }(k-1),\;{\text{u}}_{\alpha }(k-1),\text{ \textasciitilde{}}\omega_{\text{e}}(k-1)sin\left(\theta_{\text{e}}(k-1)\right) \end{array}\;\;\right]}^{T}\\ \varphi_{\beta }\left(k\right)={\left[\begin{array}{c} @ccc@i_{\beta }(k-1),\text{\textasciitilde{}}u_{\beta }(k-1),\text{\textasciitilde{}}-\omega_{\text{e}}(k-1)cos\left(\theta_{\text{e}}(k-1)\right) \end{array}\right]}^{T} \end{array} \end{array}$$ (10)

where ***φ***_α_(*k*)∈ℝ^3×1^, ***φ***_β_(*k*)∈ℝ^3×1^.

The corresponding unknown parameter vectors to be identified are:


$$\begin{array}{c} \begin{array}{c} \eta_{\alpha }\left(k\right)={\left[\begin{array}{c} @ccc@a^{\alpha }\left(k\right),\;b^{\alpha }\left(k\right),\;c^{\alpha }\left(k\right) \end{array}\;\;\right]}^{T}\\ \eta_{\beta }\left(k\right)={\left[\begin{array}{c} @ccc@a^{\beta }\left(k\right),\;b^{\beta }\left(k\right),\;c^{\beta }\left(k\right) \end{array}\;\;\right]}^{T} \end{array} \end{array}$$ (11)

with ***η***_α_(*k*)∈ℝ^3×1^ and ***η***_β_(*k*)∈ℝ^3×1^. Consequently, the estimated current based on the NARX model in the α-β stationary reference frame is formulated as:


$$\begin{array}{c} \begin{array}{c} {\hat{i}}_{\alpha }\left(k\right)=\varphi_{\alpha }^{T}\left(k\right)\eta_{\alpha }\left(k-1\right)\\ {\hat{i}}_{\beta }\left(k\right)=\varphi_{\beta }^{T}\left(k\right)\eta_{\beta }\left(k-1\right) \end{array} \end{array}$$ (12)

the parameter vectors ***η***_α_(*k*) and ***η***_β_(*k*) are updated online using the ARLS algorithm.

### 3.2. ARLS with an adaptive forgetting factor

The traditional RLS algorithm employs a fixed forgetting factor *λ*, which poses a fundamental trade-off between steady-state accuracy and dynamic response speed. To address this, an adaptive forgetting factor mechanism is designed. The factor *λ*(*k*) is adjusted online according to the estimation error:


$$\begin{array}{c} \begin{array}{c} \lambda \left(k\right)=\lambda_{\text{base}}-(\lambda_{\text{base}}-\lambda_{min})\cdot \xi \left(k\right)\\ \xi \left(k\right)=1-exp\left[-{\left(\frac{e\left(k\right)}{\delta_{\text{1}}}\right)}^{\text{2}}-{\left(\frac{\Delta e\left(k\right)}{\delta_{\text{2}}}\right)}^{\text{2}}\right]\\ \Delta e\left(k\right)=\frac{\text{1}}{N}\sum_{i=1}^{N-1}\left|e(k-i)-e(k-i-1)\right| \end{array} \end{array}$$ (13)

where 0 < *λ*_min_ < *λ*_base_ ≤ 1. Here, *e*(*k*) represents the current estimation error defined in Eq (9), and Δ*e*(*k*) quantifies the recent fluctuation level of the error, calculated as the mean absolute difference over a window of *N* past samples. The parameters *δ*_1_ and *δ*_2_ are predefined thresholds. This design ensures an exponential adaptation of *λ*(*k*) between *λ*_base_ and *λ*_min_.

**Fig 1:**
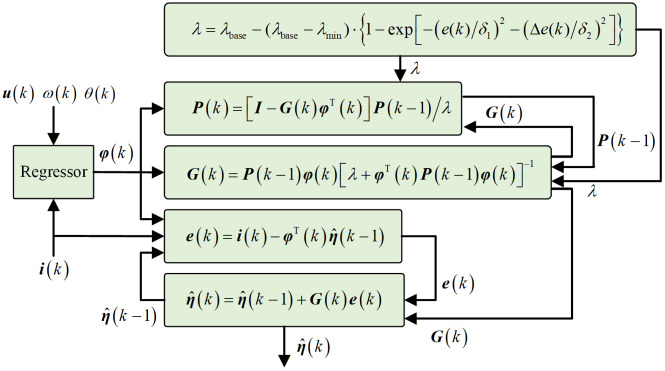
Schematic of the ARLS algorithm with a variable forgetting factor.

When a sudden load change occurs, the estimation error *e*(*k*) and its fluctuation Δ*e*(*k*) increase. This causes the forgetting factor *λ*(*k*) to decrease rapidly toward *λ*_min_, thereby accelerating the discounting of past data and enabling fast parameter tracking. Conversely, during steady-state operation, Δ*e*(*k*) approaches zero, allowing *λ*(*k*) to return to *λ*_base_. In this regime, a large *λ*(*k*) increases the effective memory horizon, thereby utilizing more historical data to suppress noise and improve estimation accuracy. The schematic diagram of the ARLS algorithm is shown in Fig 1.

### 3.3. Design of MFPC control law

The control objective is to minimize the current tracking error. The corresponding cost function is defined as:


$$\begin{array}{c} g={\left[i_{\alpha }^{*}\left(k+1\right)-{\hat{i}}_{\alpha }\left(k+1\right)\right]}^{\text{2}}+{\left[i_{\beta }^{*}\left(k+1\right)-{\hat{i}}_{\beta }\left(k+1\right)\right]}^{\text{2}} \end{array}$$ (14)

where *i*_α_^*^(*k* + 1) and *i*_β_^*^(*k* + 1) are the reference currents for the α – and β -axis, respectively.

The predicted currents are obtained from the following NARX model:


$$\begin{array}{c} \begin{array}{c} {\hat{i}}_{\alpha }\left(k+1\right)=a^{\alpha }i_{\alpha }\left(k\right)+b^{\alpha }u_{\alpha }\left(k\right)+c^{\alpha }\omega_{\text{e}}\left(k\right)sin\left(\theta_{\text{e}}\left(k\right)\right)\\ {\hat{i}}_{\beta }\left(k+1\right)=a^{\beta }i_{\beta }\left(k\right)+b^{\beta }u_{\beta }\left(k\right)+c^{\beta }\omega_{\text{e}}\left(k\right)cos\left(\theta_{\text{e}}\left(k\right)\right) \end{array} \end{array}$$ (15)

**Fig 2:**
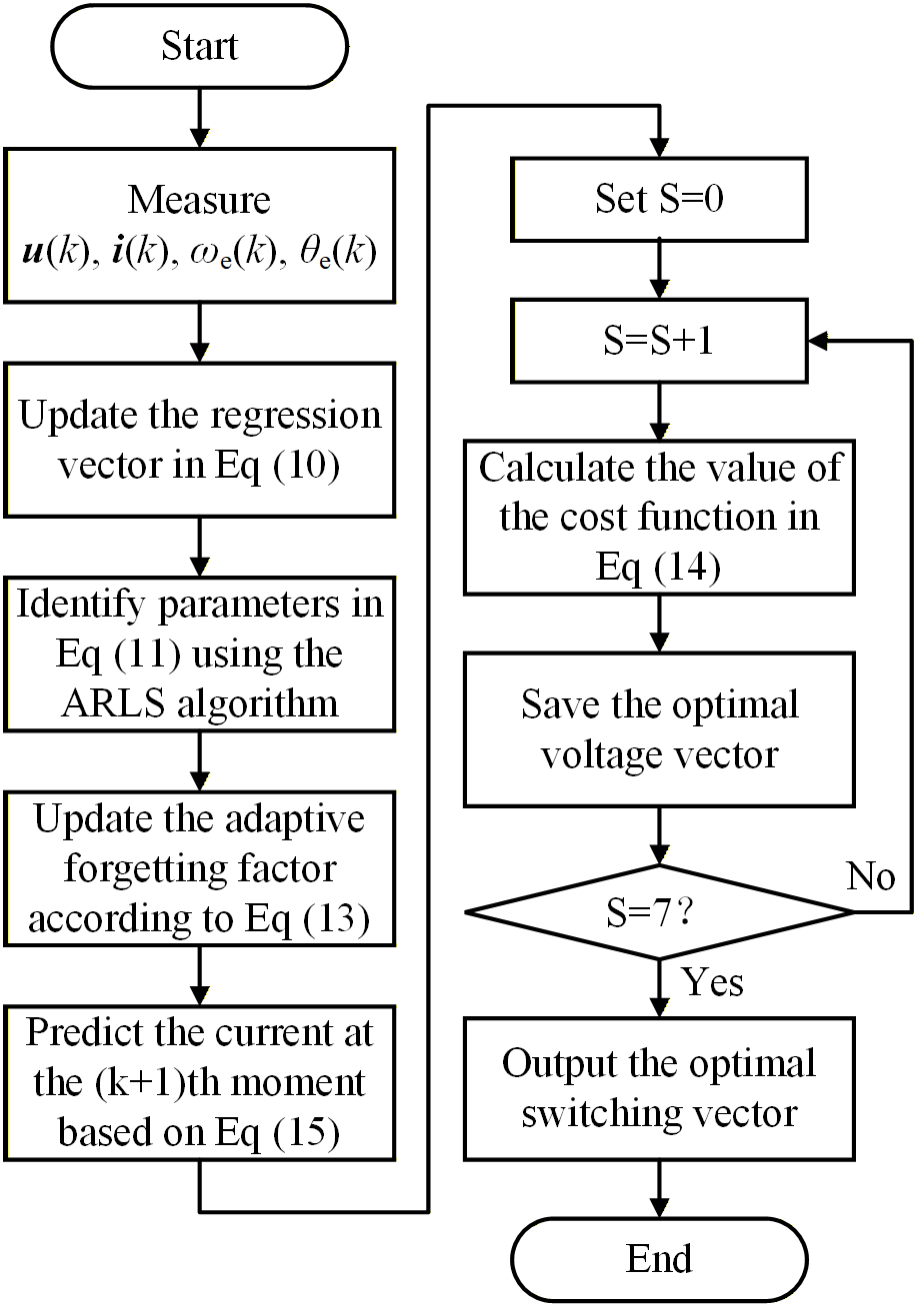
Flowchart of the proposed NARX-ARLS-MFPC algorithm.

**Fig 3:**
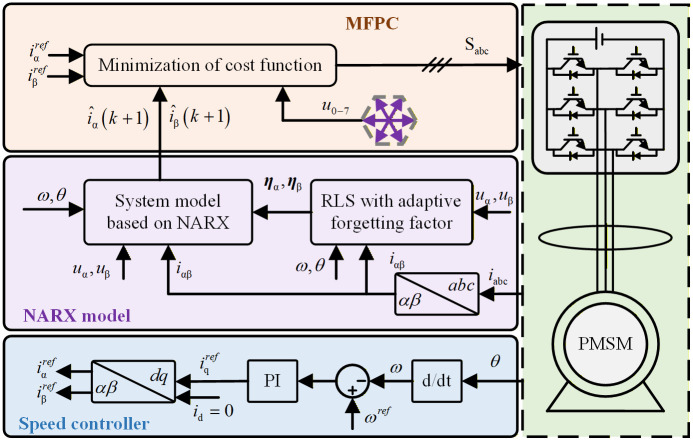
Block diagram of the NARX-ARLS-MFPC drive system for PMSM.

In each control period, the cost function g is evaluated for all candidate voltage vectors listed in Table 2. The voltage vector that minimizes g is selected as the optimal control action and applied to the inverter. The overall control flowchart and system block diagram of the proposed NARX-ARLS-MFPC strategy are shown in Fig 2 and Fig 3, respectively.

**Table 2:** Switching states and voltage vectors of the two-level inverter.

S_abc_	Voltage vector	S_abc_	Voltage vector
000	$u_{\text{0}}=0$	011	$u_{\text{4}}=-\frac{\text{2}}{\text{3}}U_{dc}$
100	$u_{\text{1}}=\frac{\text{2}}{\text{3}}U_{dc}$	001	$u_{\text{5}}=\left(-\frac{\text{1}}{\text{3}}-\text{j}\frac{\sqrt{\text{3}}}{\text{3}}\right)U_{dc}$
110	$u_{\text{2}}=\left(\frac{\text{1}}{\text{3}}+\text{j}\frac{\sqrt{\text{3}}}{\text{3}}\right)U_{dc}$	101	$u_{\text{6}}=\left(\frac{\text{1}}{\text{3}}-\text{j}\frac{\sqrt{\text{3}}}{\text{3}}\right)U_{dc}$
010	$u_{\text{3}}=\left(-\frac{\text{1}}{\text{3}}+\text{j}\frac{\sqrt{\text{3}}}{\text{3}}\right)U_{dc}$	111	$u_{\text{7}}=0$

Note: *U*_*dc*_ is the DC-bus voltage.

### 3.4. Stability analysis

Consider the following candidate Lyapunov function:


$$\begin{array}{c} V\left(k\right)=\frac{\text{1}}{\text{2}}\left[e_{\alpha }^{\text{2}}\left(k\right)+e_{\beta }^{\text{2}}\left(k\right)\right] \end{array}$$ (16)

where *e*_α_(*k*) and *e*_β_(*k*) are the current tracking errors defined as:


$$\begin{array}{c} \left\{ \begin{array}{c} @l@e_{\alpha }\left(k\right)=i_{\alpha }^{*}\left(k\right)-i_{\alpha }\left(k\right)\\ e_{\beta }\left(k\right)=i_{\beta }^{*}\left(k\right)-i_{\beta }\left(k\right) \end{array}\right. \end{array}$$ (17)

From Eq (16), it is clear that *V*(*k*) ≥ 0 for all *k*, and *V*(*k*) = 0 if and only if *e*_α_(*k*) = *e*_β_(*k*) = 0.

Supposing the current prediction error is


$$\begin{array}{c} \left\{ \begin{array}{c} @c@{\hat{e}}_{\alpha }\left(k+1\right)=i_{\alpha }^{*}\left(k+1\right)-{\hat{i}}_{\alpha }\left(k+1\right)\\ {\hat{e}}_{\beta }\left(k+1\right)=i_{\beta }^{*}\left(k+1\right)-{\hat{i}}_{\beta }\left(k+1\right) \end{array}\right. \end{array}$$ (18)

where the prediction error is defined as the deviation between model predictions and actual outputs:


$$\begin{array}{c} \left\{ \begin{array}{c} @c@\varepsilon_{\alpha }\left(k+1\right)={\hat{e}}_{\alpha }\left(k+1\right)-e_{\alpha }\left(k+1\right)\\ \varepsilon_{\beta }\left(k+1\right)={\hat{e}}_{\beta }\left(k+1\right)-e_{\beta }\left(k+1\right) \end{array}\right. \end{array}$$ (19)

Assumption 1. The NARX model identified online by the ARLS algorithm provides a sufficiently accurate one-step-ahead prediction of the stator currents. We assume there exists a positive constant *ε* > 0 such that for all time step *k*:


$$\begin{array}{c} \left\{ \begin{array}{c} @c@\left|\varepsilon_{\alpha }\left(k\right)\right|\leq \varepsilon \\ \left|\varepsilon_{\beta }\left(k\right)\right|\leq \varepsilon \end{array}\right. \end{array}$$ (20)

This assumption is a widely adopted premise in robust and adaptive control, grounded in the inherently bounded nature of physical quantities in the system: in a practical PMSM drive, the supply voltages, load torques, and rotor dynamics are all subject to physical limits. Consequently, the lumped disturbance, which aggregates unmodeled dynamics, parameter uncertainties, and external disturbances, is physically bounded. Crucially, it is the existence of a finite bound—rather than knowledge of its exact magnitude—that matters for the analysis. This boundedness condition then provides the basis for the subsequent Lyapunov-based stability analysis, where it is shown that the closed-loop system remains uniformly ultimately bounded for any admissible disturbance respecting this bound. Hence


$$\begin{array}{c} \varepsilon_{\alpha }^{\text{2}}+\varepsilon_{\beta }^{\text{2}}\leq 2\varepsilon^{\text{2}} \end{array}$$ (21)

The control voltage is selected by minimizing the sum of squared prediction errors in the following equation.


$$\begin{array}{c} u^{*}\left(k\right)=arg\underset{u\left(k\right)}{min}\left[{\hat{e}}_{\alpha }^{\text{2}}\left(k+1\right)+{\hat{e}}_{\beta }^{\text{2}}\left(k+1\right)\right] \end{array}$$ (22)

The selection ensures that the resulting prediction errors do not exceed the current tracking errors, as expressed in Eq (23).


$$\begin{array}{c} {\hat{e}}_{\alpha }^{\text{2}}\left(k+1\right)+{\hat{e}}_{\beta }^{\text{2}}\left(k+1\right)\leq e_{\alpha }^{\text{2}}\left(k\right)+e_{\beta }^{\text{2}}\left(k\right) \end{array}$$ (23)

The voltage vector selected by Eq (23) achieves local optimality in terms of the predicted tracking error based on the current adaptive NARX model. Although transient model inaccuracies can degrade instantaneous performance, the integrated ARLS estimator ensures rapid model convergence, enabling the predictive controller to recover optimal performance swiftly. The forward difference of the Lyapunov function is:


$$\begin{array}{c} \begin{array}{c} \Delta V\left(k\right)=V\left(k+1\right)-V\left(k\right)\\ =\frac{\text{1}}{\text{2}}\left[e_{\alpha }^{\text{2}}\left(k+1\right)+e_{\beta }^{\text{2}}\left(k+1\right)\right]-\frac{\text{1}}{\text{2}}\left[e_{\alpha }^{\text{2}}\left(k\right)+e_{\beta }^{\text{2}}\left(k\right)\right] \end{array} \end{array}$$ (24)

From Eq (19), we obtain:


$$\begin{array}{c} \left\{ \begin{array}{c} @l@e_{\alpha }\left(k+1\right)={\hat{e}}_{\alpha }\left(k+1\right)-\varepsilon_{\alpha }\left(k+1\right)\\ e_{\beta }\left(k+1\right)={\hat{e}}_{\beta }\left(k+1\right)-\varepsilon_{\beta }\left(k+1\right) \end{array}\right. \end{array}$$ (25)

Summing the squares:


$$\begin{array}{c} \begin{array}{cc} e_{\alpha }^{\text{2}}\left(k+1\right)+e_{\beta }^{\text{2}}\left(k+1\right)=\;\;\;\; & {\hat{e}}_{\alpha }^{\text{2}}\left(k+1\right)+{\hat{e}}_{\beta }^{\text{2}}\left(k+1\right)\\ & -2\left[{\hat{e}}_{\alpha }\left(k+1\right)\varepsilon_{\alpha }\left(k+1\right)+{\hat{e}}_{\beta }\left(k+1\right)\varepsilon_{\beta }\left(k+1\right)\right]\\ & +\varepsilon_{\alpha }^{\text{2}}\left(k+1\right)+\varepsilon_{\beta }^{\text{2}}\left(k+1\right) \end{array} \end{array}$$ (26)

Combining Eq (23) and Eq (26) yields:


$$\begin{array}{c} \begin{array}{cc} e_{\alpha }^{\text{2}}\left(k+1\right)+e_{\beta }^{\text{2}}\left(k+1\right)\leq \;\;\;\; & e_{\alpha }^{\text{2}}\left(k\right)+e_{\beta }^{\text{2}}\left(k\right)\\ \;\;\;\; & -2\left[{\hat{e}}_{\alpha }\left(k+1\right)\varepsilon_{\alpha }\left(k+1\right)+{\hat{e}}_{\beta }\left(k+1\right)\varepsilon_{\beta }\left(k+1\right)\right]\\ \;\;\;\; & +\varepsilon_{\alpha }^{\text{2}}\left(k+1\right)+\varepsilon_{\beta }^{\text{2}}\left(k+1\right) \end{array} \end{array}$$ (27)

For any real numbers a,b, we have −2ab≤2|a||b|. Therefore:


$$\begin{array}{c} -2\left[{\hat{e}}_{\alpha }\left(k+1\right)\varepsilon_{\alpha }\left(k+1\right)+{\hat{e}}_{\beta }\left(k+1\right)\varepsilon_{\beta }\left(k+1\right)\right]\leq 2\left[\left|{\hat{e}}_{\alpha }\left(k+1\right)\right|\left|\varepsilon_{\alpha }\left(k+1\right)\right|+\left|{\hat{e}}_{\beta }\left(k+1\right)\right|\left|\varepsilon_{\beta }\left(k+1\right)\right|\right] \end{array}$$ (28)

From Eq (27) and Eq (28), we obtain:


$$\begin{array}{c} \begin{array}{cc} e_{\alpha }^{\text{2}}\left(k+1\right)+e_{\beta }^{\text{2}}\left(k+1\right)\leq \;\;\; & e_{\alpha }^{\text{2}}\left(k\right)+e_{\beta }^{\text{2}}\left(k\right)\\ \;\;\; & 2\left[\left|{\hat{e}}_{\alpha }\left(k+1\right)\right|\left|\varepsilon_{\alpha }\left(k+1\right)\right|+\left|{\hat{e}}_{\beta }\left(k+1\right)\right|\left|\varepsilon_{\beta }\left(k+1\right)\right|\right]\\ \;\;\; & +\varepsilon_{\alpha }^{\text{2}}\left(k+1\right)+\varepsilon_{\beta }^{\text{2}}\left(k+1\right) \end{array} \end{array}$$ (29)

Applying the Cauchy-Schwarz inequality:


$$\begin{array}{c} \left|{\hat{e}}_{\alpha }\left(k+1\right)\right|\left|\varepsilon_{\alpha }\left(k+1\right)\right|+\left|{\hat{e}}_{\beta }\left(k+1\right)\right|\left|\varepsilon_{\beta }\left(k+1\right)\right|\leq \sqrt{{\hat{e}}_{\alpha }^{\text{2}}\left(k+1\right)+{\hat{e}}_{\beta }^{\text{2}}\left(k+1\right)}\cdot \sqrt{\varepsilon_{\alpha }^{\text{2}}\left(k+1\right)+\varepsilon_{\beta }^{\text{2}}\left(k+1\right)} \end{array}$$ (30)

From the control law and Assumption 1, we obtain:


$$\begin{array}{c} \left\{ \begin{array}{c} @l@\sqrt{{\hat{e}}_{\alpha }^{\text{2}}\left(k+1\right)+{\hat{e}}_{\beta }^{\text{2}}\left(k+1\right)}\leq \sqrt{e_{\alpha }^{\text{2}}\left(k\right)+e_{\beta }^{\text{2}}\left(k\right)}\\ \sqrt{\varepsilon_{\alpha }^{\text{2}}\left(k+1\right)+\varepsilon_{\beta }^{\text{2}}\left(k+1\right)}\leq \sqrt{\text{2}}\varepsilon \end{array}\right. \end{array}$$ (31)

From Eq (29), Eq (30), and Eq (31), we obtain:


$$\begin{array}{c} e_{\alpha }^{\text{2}}\left(k+1\right)+e_{\beta }^{\text{2}}\left(k+1\right)\leq e_{\alpha }^{\text{2}}\left(k\right)+e_{\beta }^{\text{2}}\left(k\right)+2\sqrt{\text{2}}\varepsilon \sqrt{e_{\alpha }^{\text{2}}\left(k\right)+e_{\beta }^{\text{2}}\left(k\right)}+2\varepsilon^{\text{2}} \end{array}$$ (32)

Substituting Eq (31) into Δ*V*(*k*):


$$\begin{array}{c} \Delta V\left(k\right)\leq \sqrt{\text{2}}\varepsilon \sqrt{e_{\alpha }^{\text{2}}\left(k\right)+e_{\beta }^{\text{2}}\left(k\right)}+\varepsilon^{\text{2}} \end{array}$$ (33)

From Eq (16) and Eq (33), we obtain:


$$\begin{array}{c} \Delta V\left(k\right)\leq 2\varepsilon \sqrt{V\left(k\right)}+\varepsilon^{\text{2}} \end{array}$$ (34)

Provided the model prediction error is bounded (*ε* < ∞) and the increment of the Lyapunov function satisfies Eq (34), the system’s tracking error energy *V*(*k*) is guaranteed to be bounded. Consequently, the closed-loop system is uniformly ultimately bounded. In the ideal case of an accurate prediction model (*ε* = 0), it follows that Δ*V*(*k*) ≤ 0, which implies the system is Lyapunov stable.

### 3.5. Analysis of computational complexity and real-time feasibility

The practical implementation of the proposed NARX-ARLS-MFPC necessitates an analysis of its computational cost and real-time feasibility, which are critical for deployment on embedded processors in drive systems.

The computational load within each control cycle stems from two sequential stages: online parameter identification and optimal vector selection. The identification employs the ARLS algorithm to update two independent 3-dimensional parameter vectors ***η***_α_(*k*) and ***η***_β_(*k*). The dominant cost is the update of a 3 × 3 covariance matrix ***P***_α_(*k*) and ***P***_β_(*k*), involving matrix-vector multiplications and an outer product, with a fixed complexity of *O*(*n*^2^) where *n* = 3. Subsequently, the control law adopts FCS-MPC strategy, evaluating the cost function Eq (14) for all 8 candidate voltage vectors listed in Table 2. For each vector, this requires one-step current prediction using Eq (15), constituting a few scalar multiplications and additions. While the FCS scheme necessitates evaluating all 8 vectors, the operations for each are independent and lightweight compared to the matrix update.

Covariance matrix update size: the core of the ARLS identification is the recursive update of a 3 × 3 symmetric covariance matrix for each axis. The algorithm operates on this fixed, small-dimensional matrix.

Feasibility at typical PMSM control frequencies (e.g., 10–20 kHz): the complete control cycle comprises fixed, non-iterative operations with a predictable worst-case execution time. The most computationally intensive task is the 3 × 3 matrix update, a routine efficiently handled by the arithmetic units of modern motor control DSPs (e.g., TI C2000 series). Empirical measurements within the MATLAB/Simulink simulation environment confirm that the core 3 × 3 matrix update requires approximately 0.25 µs per call. This simulated execution time demonstrates the fundamental computational efficiency of the algorithm and strongly indicates its real-time feasibility for implementation on embedded processors. In this study, the complete algorithm was successfully executed within a stringent 10 µs (100 kHz) simulation cycle, which is significantly shorter than 50–100 µs (10–20 kHz) cycle typical of industrial drives. This confirms that the proposed method possesses ample time margin for real-time implementation on common hardware. The choice of the compact third-order NARX model is pivotal to this feasibility, achieving an effective balance between model expressiveness and computational tractability.

## 4. Simulation results and discussion

### 4.1. Simulation and implementation details

The comparative performance presented in subsections 4.2, 4.3 and 4.4 were generated using a simulation model developed in MATLAB/Simulink 2018b. The FCS-MPC is implemented to directly output the optimal inverter switching states without a carrier-based modulation stage. For the performance evaluation in subsections 4.2, 4.3.1 and 4.4, the rotor speed and position are assumed to be measured by ideal sensors. The analysis of robustness under variable noise levels is presented separately in subsection 4.3.2. All critical parameters for the motor and the control algorithms are provided in Table 3, ensuring the reproducibility of the presented simulations.

**Table 3:** Simulation parameters.

Parameter	Value	Parameter	Value
DC-bus voltage	300 V	*λ*_base_	1
*Ψ*_f_	0.1827 Wb	*λ*_min_	0.96
*L*_s_	0.00525 H	*δ*_1_	0.9
*R*_s_	0.9585 Ω	*δ*_2_	0.5
Control sampling period	10 μs	*N*	10

### 4.2. Model selection and comparison

To determine the optimal model for the NARX-ARLS-MFPC, three different model structures are evaluated and compared. The mathematical formulations of the evaluated models are listed in Table 4.

**Table 4:** Models.

	Model 1	Model 2	Model 3
Regression vectors	***φ***_α_(*k*) = [*i*_α_(*k*-1), *u*_α_(*k*-1)]^*T*^ ***φ***_β_(*k*) = [*i*_β_(*k*-1), *u*_β_(*k*-1)]^*T*^	***φ***_α_(*k*) = [*i*_α_(*k*-1), *i*_α_(*k*-2), *u*_α_(*k*-1), *ω*_e_(*k*-1)sin(*θ*_e_(*k*-1))]^*T*^ ***φ***_β_(*k*) = [*i*_β_(*k*-1), *i*_β_(*k*-2), *u*_β_(*k*-1), -*ω*_e_(*k*-1)cos(*θ*_e_(*k*-1))]^*T*^	***φ***_α_(*k*) = [*i*_α_(*k*-1), *u*_α_(*k*-1), *ω*_e_(*k*-1)sin(*θ*_e_(*k*-1))]^*T*^ ***φ***_β_(*k*) = [*i*_β_(*k*-1), *u*_β_(*k*-1), -*ω*_e_(*k*-1)cos(*θ*_e_(*k*-1))]^*T*^
Parameter vectors	***η***_α_(*k*) = [*a*^α^(*k*), *b*^α^(*k*)]^*T*^ ***η***_β_(*k*) = [*a*^β^(*k*), *b*^β^(*k*)]^*T*^	***η***_α_(*k*) = [*a*^α^(*k*), *b*^α^(*k*), *c*^α^(*k*), *d*^α^(*k*)]^*T*^ ***η***_β_(*k*) = [*a*^β^(*k*), *b*^β^(*k*), *c*^β^(*k*), *d*^β^(*k*)]^*T*^	***η***_α_(*k*) = [*a*^α^(*k*), *b*^α^(*k*), *c*^α^(*k*)]^*T*^ ***η***_β_(*k*) = [*a*^β^(*k*), *b*^β^(*k*), *c*^β^(*k*)]^*T*^

The comparative analysis employs four key performance indicators—RMS current tracking errors (both iα and iβ), switching frequency, and THD—to comprehensively assess each model’s capabilities. Table 5 presents the performance comparison of the three models across four rotor speeds: 150 r/min (10% of rated speed), 750 r/min (50%), 1500 r/min (100%), and 1800 r/min (120%).

**Table 5:** Performance comparison of three models.

Model	RMS(*i*_α_ tracking error) (A)				RMS(*i*_β_ tracking error) (A)				Switching frequency (kHz)				THD (%)			
	150	750	1500	1800	150	750	1500	1800	150	750	1500	1800	150	750	1500	1800
1	0.027	0.053	0.102	0.111	0.019	0.011	0.011	0.011	4.729	19.439	17.237	14.778	8.16	8.14	8.09	7.97
2	0.024	0.106	0.203	0.221	0.011	0.010	0.012	0.013	4.729	19.423	17.638	14.784	8.17	8.30	8.31	8.16
3	0.012	0.038	0.039	0.039	0.005	0.005	0.006	0.007	6.409	19.603	18.461	14.914	7.84	7.72	8.09	7.58

Based on the performance comparison in Table 5, Model 3 is identified as the optimal structure as it achieves the best overall performance across the wide speed range. It consistently delivers the lowest RMS current tracking errors at all operating speeds, for example, maintaining the *i*_α_ error between 0.012 A and 0.039 A and the *i*_β_ error between 0.005 A and 0.007 A. Furthermore, it generally provides the best harmonic performance, with THD values between 7.58% and 8.09%. This performance consistently outperforms both the simpler Model 1 and the more complex Model 2, especially at medium to high speeds. Although Model 3 operates at a marginally higher switching frequency, the substantial and consistent improvements in tracking precision and waveform quality across the entire operating range justify this minor increase. Therefore, Model 3 provides the most effective representation of the system dynamics for the proposed NARX-ARLS-MFPC strategy.

### 4.3. Performance under inductance and flux mismatches

#### 4.3.1. Case 1: without noise.

The control strategies utilize the nominal motor parameters *R*_s_, *L*_s_, and *Ψ*_f_. To simulate parameter mismatches, the actual motor parameters in the simulation are set to different values *R*_sa_, *L*_sa_, and *Ψ*_fa_. This subsection evaluates robustness under inductance and flux linkage mismatch conditions.

**Fig 4:**
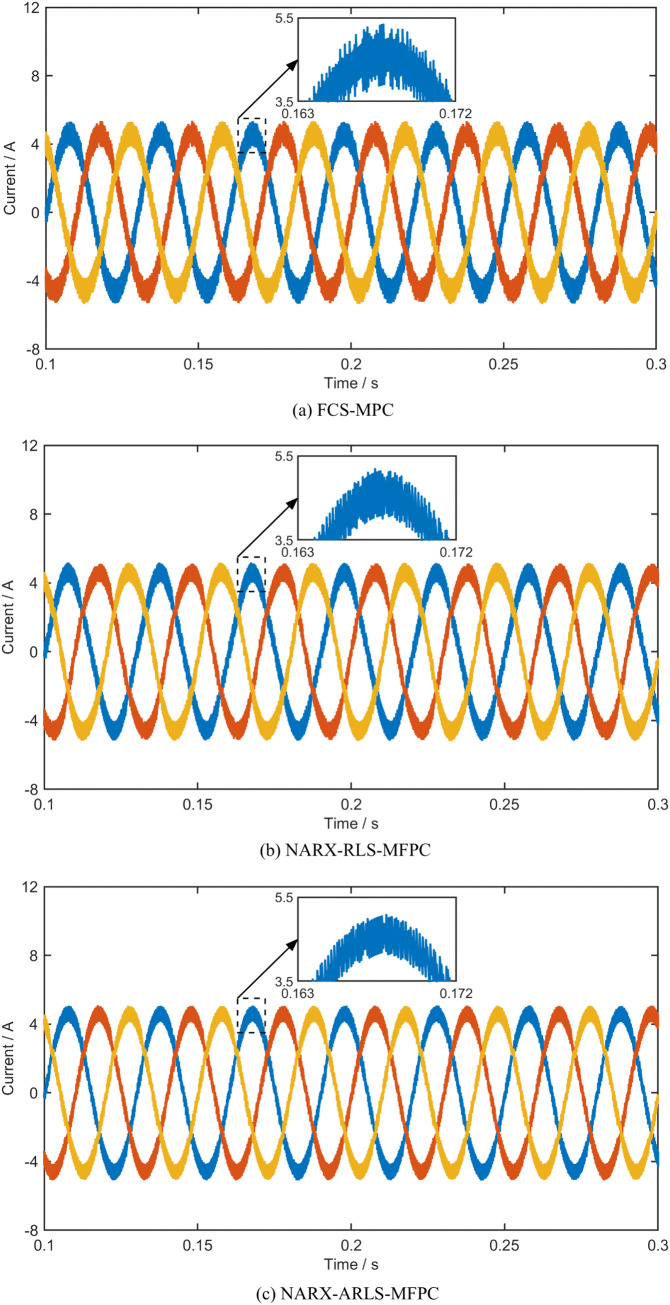
Three-phase current responses under *L*_sa_/*L*_s_  = 0.4 and *Ψ*_fa_/*Ψ*_f_ = 0.4. (a) FCS-MPC, (b) NARX-RLS-MFPC, (c) NARX-ARLS-MFPC.

**Fig 5:**
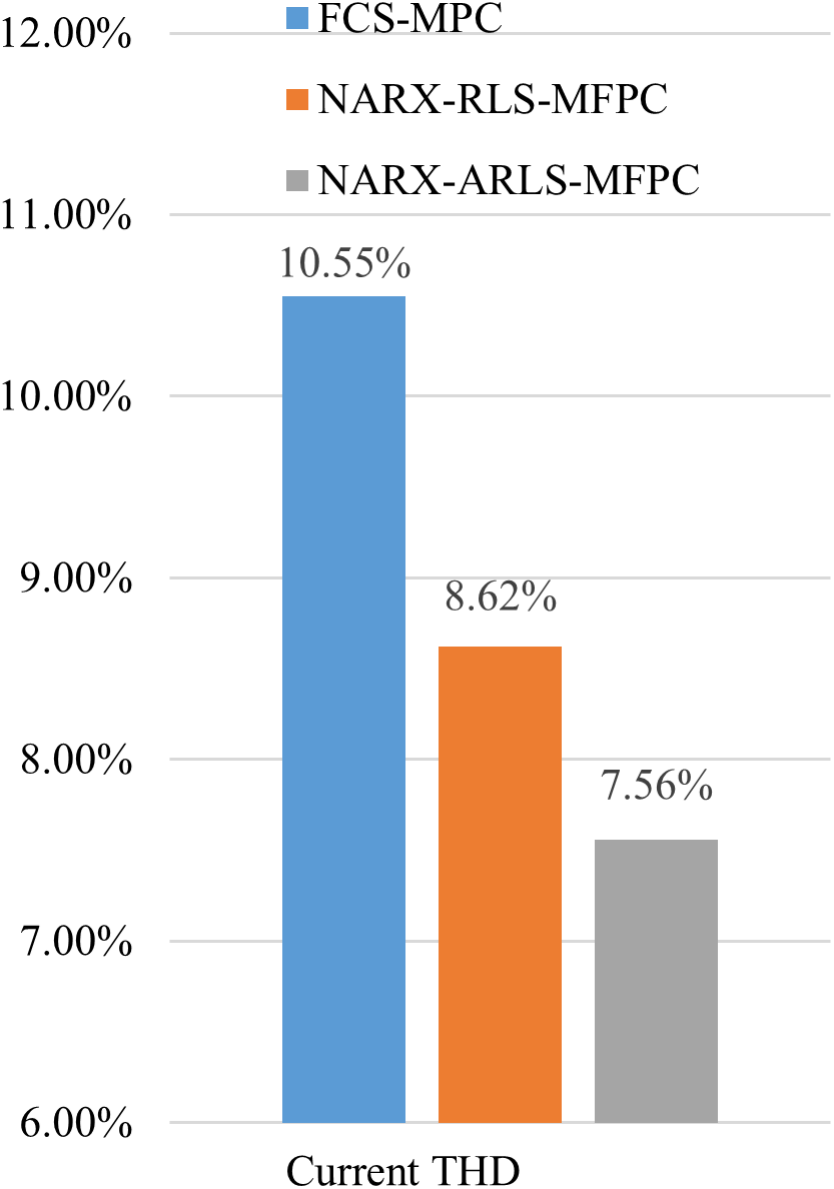
Three-phase current THD values under *L*_sa_/*L*_s_ = 0.4 and *Ψ*_fa_/*Ψ*_f_ = 0.4.

Fig 4 shows the three-phase current responses under FCS-MPC, NARX-RLS-MFPC, and the proposed NARX-ARLS-MFPC controls, given mismatches of *L*_sa_/*L*_s_ = 0.4 and *Ψ*_fa_/*Ψ*_f_ = 0.4. The corresponding current THD values are presented in Fig 5.

As shown in Figs 4 and 5, the current quality of the FCS-MPC strategy deteriorates due to the parameter mismatches, exhibiting substantial ripples with an average THD of 10.55%. In contrast, the average THD values for NARX-RLS-MFPC and NARX-ARLS-MFPC are 8.60% and 7.56%, respectively. Therefore, compared to FCS-MPC and the baseline NARX-RLS-MFPC, the THD of the proposed NARX-ARLS-MFPC method is reduced by 28.3% and 12.3%, respectively, demonstrating its superior robustness.

#### 4.3.2. Case 2: parameter sensitivity analysis with simulated sensor noise.

To further evaluate the robustness of the proposed controller against practical measurement imperfections, a noise sensitivity study is conducted. Additive white Gaussian noise with different standard deviations (*σ* = 0.05, 0.1, 0.5) is superimposed on the measured αβ axes current signals. The performance of the proposed NARX-ARLS-MFPC under various noise levels and different internal parameter sets is summarized in Table 6.

**Table 6:** Performance under different noise levels and parameter sets.

No.	Parameter setting (*δ*₁, *δ_2_*, *N*, *λ*_min_)	THD under noise level σ = 0.05		THD under noise level σ = 0.1		THD under noise level σ = 0.5	
		Convergence	THD	Convergence	THD	Convergence	THD
1	2.0, 1.0, 100, 0.99	Converged	7.66%	Converged	8.12%	Converged	13.61%
2	1.6, 0.6, 20, 0.98	Converged	7.67%	Converged	8.12%	Converged	13.64%
3	0.8, 0.3, 10, 0.96	Converged	7.66%	Converged	8.12%	Converged	13.63%
4	0.4, 0.15, 5, 0.90	Converged	7.59%	Converged	7.95%	Converged	13.50%
5	0.2, 0.1, 3, 0.85	Converged	7.56%	Converged	8.03%	Converged	13.48%
6	0.1, 0.05, 3, 0.8	Converged	11.47%	Converged	11.80%	Converged	16.16%
7	0.05, 0.01, 3, 0.8	Diverged	–	Diverged	–	Diverged	–
8	0.01, 0.001, 2, 0.8	Diverged	–	Diverged	–	Diverged	–

For parameter Sets 1–5, the system maintains stable convergence with a THD below 8.5% across noise levels up to σ = 0.1. Set 6 maintains convergence but exhibits significantly higher THD of 11.47%−16.16%, indicating greater noise sensitivity. In contrast, Sets 7 and 8 diverge completely, demonstrating that excessively small innovation thresholds (*δ*_1_, *δ*_2_) when operating at the lower bound of effective memory length critically compromise robustness. Therefore, appropriate parameter tuning is essential for reliable operation amidst sensor noise.

The sensitivity analysis above elucidates the distinct roles of the two key parameter classes. First, the thresholds *δ*_1_ and *δ*_2_ serve as robustness gatekeepers, preventing the model from being updated with noise-corrupted data. The divergence of Sets 7 and 8 demonstrates that if the thresholds are set too small, even minor noise is mistaken for a genuine system change, triggering destructive adaptation. Therefore, *δ*_1_ and *δ*_2_ must be set above the expected noise amplitude to ensure stability. Second, the effective memory horizon of the algorithm, governed by the data window length *N* and the minimum forgetting factor *λ*_min_, determines the trade-off between tracking agility and steady-state precision. Set 6, with a relatively small *N* and low *λ*_min_, maintains convergence but exhibits degraded THD. This indicates a shortened effective memory, which increases noise susceptibility and degrades accuracy. Conversely, the superior performance of Sets 1–5 across various noise levels is attributable to a sufficiently long effective memory (achieved by a larger N or a higher *λ*_min_), which provides filtering and ensures precision.

Based on these insights, practical tuning guidelines are derived: 1) Set *δ*_1_ and *δ*_2_ conservatively based on the estimated sensor noise level to guarantee robustness; 2) Select *N* and *λ*_min_ to provide a sufficient effective memory for noise attenuation, which is critical for high-precision applications. The results identify a stable and well-performing parameter region (Sets 1–5) and distinguish it from the sensitive regions (Sets 6–8). These findings and guidelines establish a principled basis for parameter selection that ensures robustness and noise immunity. These findings empirically validate that within the mapped stable parameter region, the closed-loop behavior aligns with the uniformly ultimately bounded guarantee derived from the Lyapunov framework, thereby confirming that the heuristic tuning rules operate within the theoretically sound margin.

### 4.4. Performance under load transient and R-L parameter mismatches

#### 4.4.1. Case 1: without parameter perturbations.

**Fig 6:**
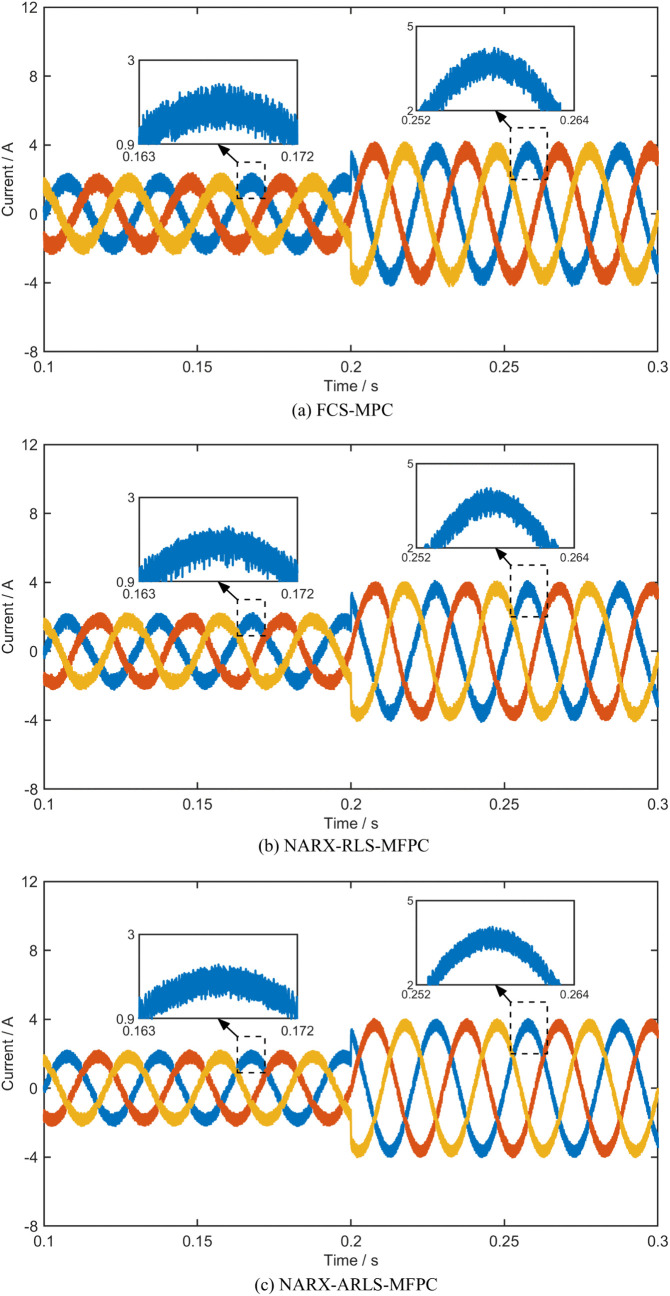
Three-phase current responses under *R*_sa_/*R*_s_ = 2 and *L*_sa_/*L*_s_ = 0.5. (a) FCS-MPC, (b) NARX-RLS-MFPC, (c) NARX-ARLS-MFPC.

**Fig 7:**
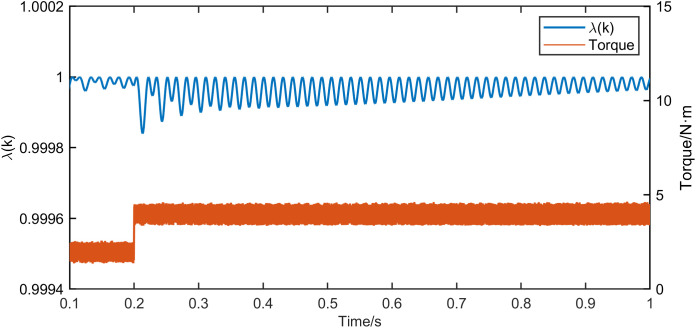
Evolution of the variable forgetting factor *λ*(*k*).

**Fig 8:**
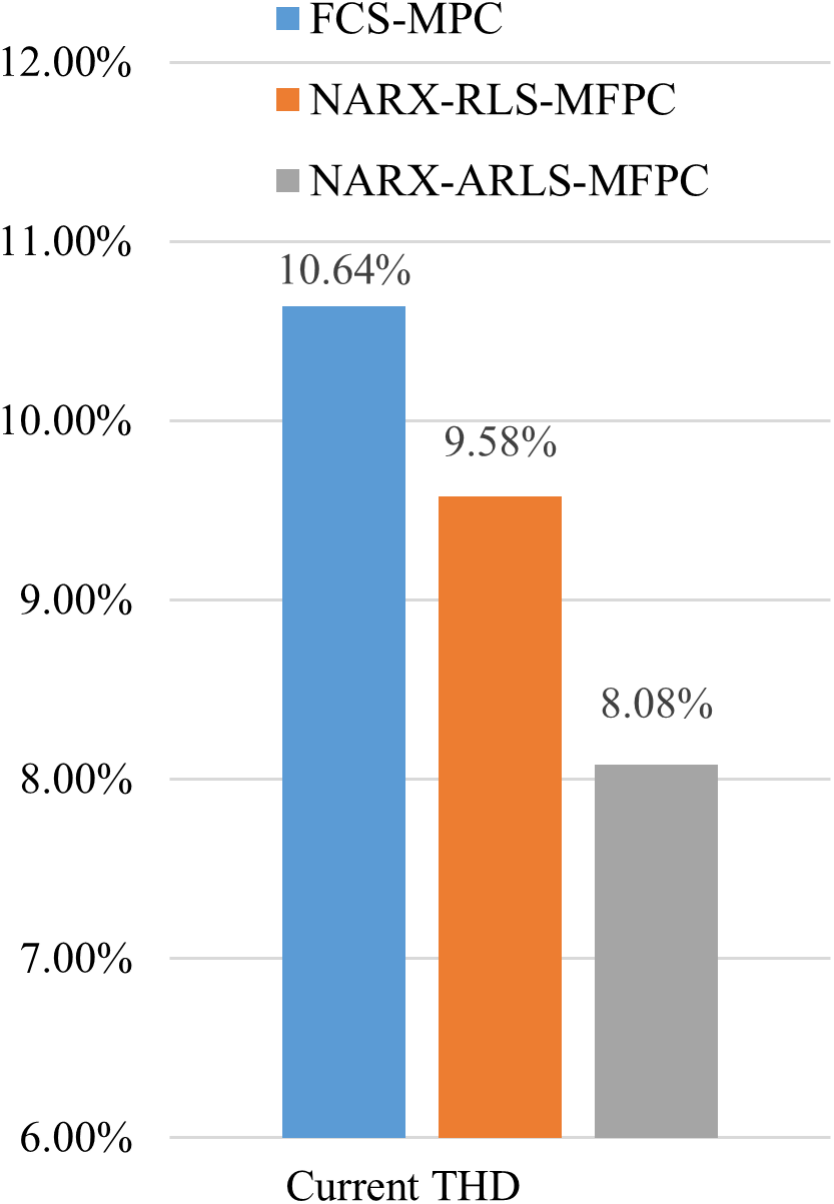
Three-phase current THD values under *R*_sa_/*R*_s_ = 2 and *L*_sa_/*L*_s_ = 0.5.

The system operates under a load torque that steps from 2 N·m to 4 N·m at 0.2 s, with parameter mismatch conditions set to *R*_sa_/*R*_s_ = 2 and *L*_sa_/*L*_s_ = 0.5. The three-phase current responses under the FCS-MPC, NARX-RLS-MFPC, and the proposed NARX-ARLS-MFPC strategies are shown in Fig 6. The evolution of the variable forgetting factor *λ*(*k*) during this load-torque step is presented in Fig 7. The current THD was measured over one cycle following the load step, as illustrated in Fig 8.

As shown in Fig 8, the THD values are 10.64% for FCS-MPC, 9.58% for NARX-RLS-MFPC, and 8.08% for NARX-ARLS-MFPC. Compared to the FCS-MPC and NARX-RLS-MFPC strategies, the proposed method reduces the THD by 24.1% and 15.7%, respectively.

#### 4.4.2. Case 2: with parameter perturbations.

To evaluate the robustness and statistical performance of the proposed and comparative control strategies under parameter uncertainties, a simulation study with 30 independent runs was conducted. In each run, the actual motor parameters were subject to both systematic mismatches and stochastic perturbations: the inductance was set to 0.5 times its rated value with a ± 20% uniformly distributed random variation, and the resistance was set to 2.0 times its rated value with a ± 20% variation of the same type. Key performance metrics—including settling time, overshoot, RMS current tracking error, torque ripple, average switching frequency, and current THD—were recorded for each run. Table 7 presents the mean and standard deviation for each metric, providing a statistically sound comparison of the algorithms’ performance under uncertainty.

**Table 7:** Performance comparison of control strategies under parameter perturbations.

Control strategies	Settling time (s)	Overshoot (%)	RMS(*i*_α_ tracking error) (A)	RMS(*i*_β_ tracking error) (A)	Torque ripple (N·m)	Switching frequency (kHz)	THD (%)
FCS-MPC	0.1997 ± 0.0002	4.09 ± 0.82	–	–	1.543 ± 0.309	37.728 ± 1.380	11.49 ± 1.95
ARX-ARLS-MFPC	0.1797 ± 0.0176	1.83 ± 0.15	0.0998 ± 0.0006	0.0278 ± 0.0118	1.049 ± 0.073	16.637 ± 1.075	8.23 ± 0.72
NARX-RLS-MFPC	0.1695 ± 0.0134	1.80 ± 0.13	0.0323 ± 0.0003	0.0155 ± 0.0040	1.065 ± 0.073	15.685 ± 0.384	8.03 ± 0.54
NARX-ARLS-MFPC	0.1536 ± 0.0047	1.56 ± 0.09	0.0325 ± 0.0005	0.0106 ± 0.0002	0.905 ± 0.060	15.254 ± 0.151	6.90 ± 0.44
ESO-based ultra-local model	0.1962 ± 0.0049	3.43 ± 0.44	–	–	1.267 ± 0.156	36.697 ± 1.212	9.96 ± 1.09

1. Ablation study on algorithmic components

To validate the contribution of each key component, an ablation study is conducted by comparing four strategies: the conventional FCS-MPC, the ARX-ARLS-MFPC which employs a linear model with adaptive forgetting, the NARX-RLS-MFPC which uses a nonlinear model with fixed forgetting, and the proposed NARX-ARLS-MFPC.

As shown in Table 7, compared to the linear ARX-ARLS-MFPC, the NARX-RLS-MFPC demonstrates the benefit of the nonlinear model by reducing the current THD and improving current tracking accuracy. Conversely, the ARX-ARLS-MFPC exhibits a marginally faster settling time, highlighting a trade-off in transient response. The proposed NARX-ARLS-MFPC, integrating both components, surpasses all other variants. It achieves the best overall performance, including the shortest settling time of 0.1536 s, the lowest overshoot of 1.56%, the lowest torque ripple of 0.905 N·m, and the lowest current THD of 6.90%, with significantly reduced standard deviations. This confirms that the synergistic integration of the nonlinear NARX model and the adaptive recursive least-squares algorithm provides enhanced robustness and superior capability in handling model mismatches and disturbances.

1. Comparison with the ESO-based ultra-local model method

To further contextualize the performance of the proposed method within contemporary research, it was benchmarked against a recent advanced model-free strategy: the extended sliding mode observer-based ultra-local model method [14].

As evidenced in Table 7, the proposed NARX-ARLS-MFPC demonstrates comprehensive advantages. It exhibits superior dynamic performance, featuring a faster settling time of 0.1536 s compared to 0.1962 s, and a lower overshoot of 1.56% compared to 3.43%. Furthermore, it delivers enhanced steady-state quality, reflected in lower torque ripple and current THD, as well as a significantly higher switching efficiency of approximately 15.254 kHz versus 36.697 kHz.

The presented simulation results demonstrate that the proposed NARX-ARLS-MFPC method, through its synergistic combination of adaptive parameter identification and nonlinear dynamic modeling, effectively suppresses current distortion caused by model mismatch and improves current quality under dynamic load conditions.

### 4.5. Discussion

The performance improvements of the proposed NARX-ARLS-MFPC stem from its two key features. First, the adaptive forgetting factor *λ*(*k*) enhances robustness against parameter mismatch. The key mechanism is a closed-loop adjustment: when a sudden load step introduces model error, the prediction innovation increases. This triggers an automatic decrease in *λ*(*k*) in Fig 7, which shortens the algorithm’s effective memory window and accelerates parameter updates to correct the model. Once the parameters converge to the new condition, *λ*(*k*) rises, strengthening memory and noise immunity. This dynamic balance between learning speed and estimation stability underpins the consistent tracking performance under varying conditions, as evidenced in Fig 6. Second, the inclusion of nonlinear NARX terms is critical for accuracy at high speeds, where the back-EMF introduces a speed‑dependent nonlinearity. The NARX model explicitly includes cross-terms that directly mirror physical coupling. By doing so, the predictor inherently accounts for the back-EMF effect, leading to more accurate current predictions. This explains the significantly reduced prediction error and improved torque control accuracy of the proposed method at high speeds.

The performance of the proposed method is subject to several practical limits. First, at extremely low rotational speeds, the back-EMF amplitude diminishes sharply, resulting in insufficient persistent excitation for the ARLS identifier. Consequently, online parameter adaptation loses asymptotic convergence, and the identifier cannot accurately track slow time-varying parameter deviations. It should be emphasized that insufficient persistent excitation only deteriorates parameter convergence and control transient performance, rather than jeopardizing closed-loop stability. The Lyapunov stability proof underlying Inequality (23) only requires the overall estimation error to be bounded, not asymptotic convergence of parameters. For the ARLS with a forgetting factor, its classical uniformly ultimate boundedness property [22] guarantees that the parameter estimation error remains bounded even under inadequate persistent excitation, provided that the regression signals stay bounded. Owing to the linearly parameterized prediction model, bounded parameter estimation error directly leads to bounded one-step-ahead prediction error, which ensures that Assumption 1 remains satisfied throughout low-speed operation. Therefore, the stability conclusion derived from Inequality (23) remains valid over the entire low-speed region. Second, under extreme measurement noise, the innovation sequence can be corrupted, misleading the adaptation law. Finally, during rapid speed reversals, the plant dynamics change abruptly. A transient dip may occur before model re-adaptation.

Regarding hardware implementation, the proposed method features moderate computational load. Its core overhead lies in the ARLS covariance matrix update with O(n^2^) complexity and a limited number of regressors, which can be readily accommodated by common low-cost 32-bit fixed-point DSPs and ARM microcontrollers. The fixed-point C implementation eliminates dependence on floating-point units and further cuts hardware cost, making the scheme suitable for cost-sensitive industrial drive scenarios. In terms of scalability, the data-driven nature of the proposed method gives it inherent potential to be extended to PMSMs of different power ratings and operating conditions.

The next immediate step is to experimentally validate the proposed scheme on a physical PMSM drive testbed. This experimental phase will focus on three key practical directions: (1) obtaining empirical execution-time benchmarks by deploying the fixed-point C implementation of the algorithm on a low-cost real-time embedded platform; (2) investigating practical implementation issues, including performance under actual sensor noise, computational resource constraints, and the effects of inverter non-idealities; (3) verifying the scalability of the proposed data-driven framework across PMSMs of different power ratings and typical operating regimes including flux weakening, heavy load saturation, and low-speed high-torque scenarios. The findings from this experimental work will provide a comprehensive assessment of the method’s efficacy in a real-world system.

## 5. Conclusion

This paper has developed and validated a NARX-ARLS-MFPC method for PMSMs. The core of this method is the synergistic integration of a nonlinear NARX model, which accurately captures the motor’s complex dynamics, and an adaptive ARLS identifier with a variable forgetting factor, which enables real-time tracking of time-varying parameters. This integration fundamentally eliminates the dependency on precise prior knowledge of physical motor parameters. Extensive simulations have demonstrated that the proposed method robustly mitigates performance degradation caused by model mismatches. It significantly suppresses current distortion and improves current quality under a wide range of challenges, including severe parameter mismatches, sensor noise, and dynamic load transients, thereby demonstrating its superior robustness and control performance.

## Supporting information

S1 File MATLAB codes for NARX model and ARLS algorithm, simulation raw data, and schematic diagram of the PMSM control structure. (ZIP)
