# Supplementary figures and images for: Model-free predictive control for PMSM based on a nonlinear autoregressive exogenous model and an adaptive recursive least squares algorithm
Source: PLoS One. 2026 Jul 27;21(7):e0354803. doi: 10.1371/journal.pone.0354803 (PMC13405074; doi:10.1371/journal.pone.0354803)

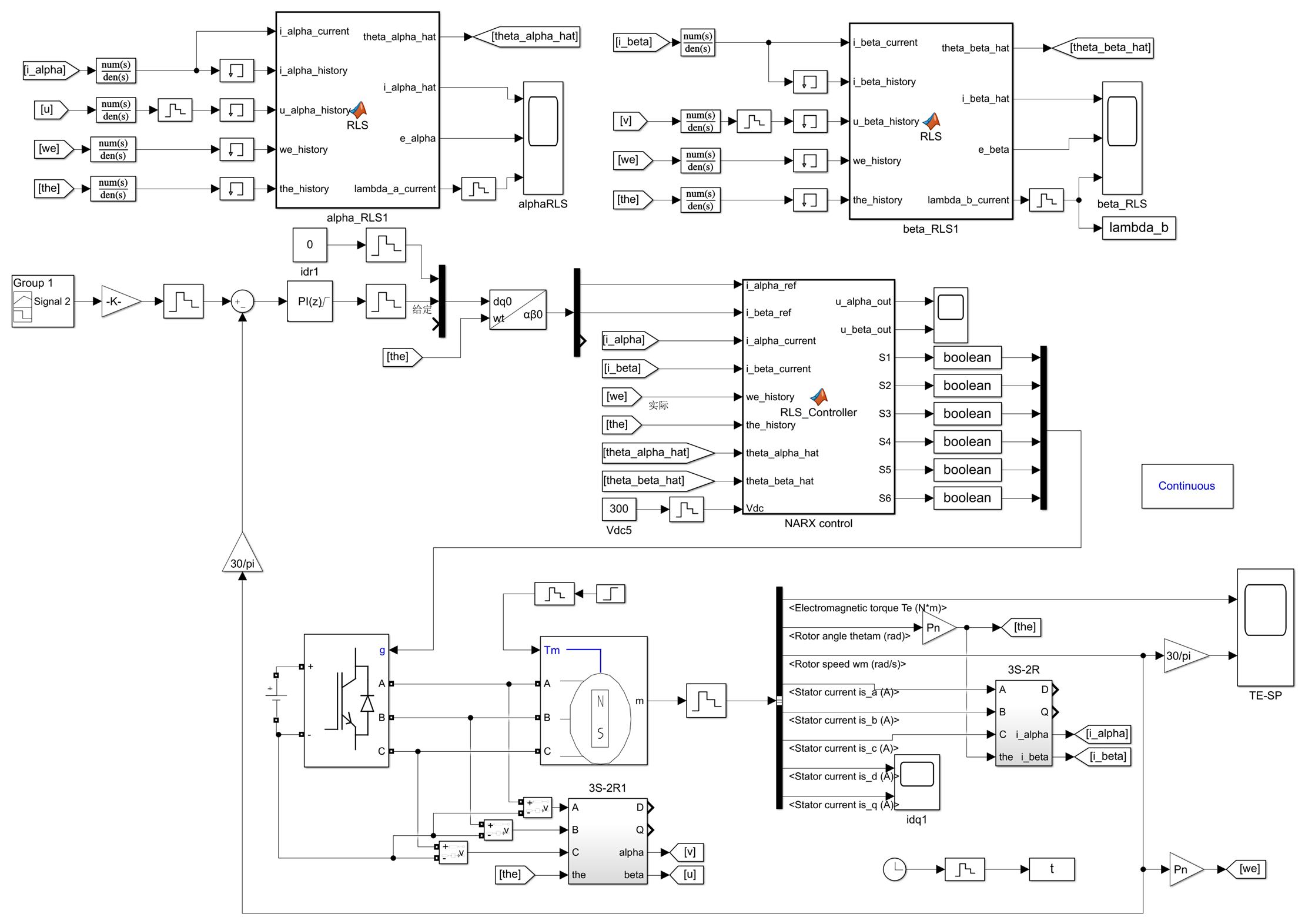

Supplement: S1 File — (ZIP) [file pone.0354803.s001.zip › Supporting Information/model.tif]
